# Low Effectiveness of Mid-Infrared Spectroscopy Prediction Models of Mediterranean Italian Buffalo Bulk Milk Coagulation Traits

**DOI:** 10.3390/foods13131957

**Published:** 2024-06-21

**Authors:** Alberto Guerra, Carlo Boselli, Tiziana Galli, Letizia Ciofi, GianLuca Fichi, Massimo De Marchi, Carmen L. Manuelian

**Affiliations:** 1 Department of Agronomy, Food, Natural Resources, Animals and Environment, University of Padova, Viale dell’Università 16, 35020 Legnaro, Italy; alberto.guerra@unipd.it (A.G.); massimo.demarchi@unipd.it (M.D.M.); 2 Experimental Zooprophylactic Institute Lazio and Toscana “Mariano Aleandri”, Via Appia Nuova 1411, 00178 Rome, Italy; carlo.boselli@izslt.it (C.B.); tiziana.galli@izslt.it (T.G.); letizia.ciofi@izslt.it (L.C.); gianluca.fichi@izslt.it (G.F.); 3 Group de Recerca en Remugats (G2R), Departament de Ciència Animal i dels Aliments, Universitat Autònoma de Barcelona, 08193 Bellaterra, Spain

**Keywords:** mid-infrared spectroscopy, bulk buffalo milk, technological properties

## Abstract

**Simple Summary:**

In Italy, buffalo milk is mainly transformed into ‘Mozzarella di Bufala Campana’, a Protected Designation of Origin (PDO) cheese. A quick method for predicting the coagulation properties of the milk before cheese production could enhance the efficiency of the industry. Therefore, the aim of this paper was to evaluate the potential use of mid-infrared spectroscopy to predict milk coagulation traits in bulk milk from Mediterranean Italian buffaloes. A total of 1736 bulk milk samples from 55 farms in central Italy were analyzed. Prediction models using mid-infrared spectroscopy were built with a modified partial least-squares regression using an external validation dataset. The best prediction model was obtained for curd firmness, but it was still inaccurate enough to replace traditional methods.

**Abstract:**

This study evaluated the potential use of mid-infrared spectroscopy to predict milk coagulation traits in bulk milk from Mediterranean Italian buffaloes. A total of 1736 bulk milk samples from 55 farms in central Italy were collected during the official milk quality testing system. The prediction models were developed based on modified partial least-squares regression with 75% of the samples and validated with the remaining samples. All bulk milk samples coagulated between 7.37 and 29.45 min. Average values for milk coagulation traits in the calibration set were 17.71 min, 3.29 min, and 38.83 mm for rennet coagulation time, curd firming time, and curd firmness, respectively. The validation set included samples with similar mean and standard deviation for each trait. The prediction models showed the greatest coefficient of determination of external validation (0.57) and the ratio of prediction to deviation (1.52) for curd firmness. Similar fitting statistics of the prediction models were obtained for rennet coagulation time and curd firming time. In conclusion, the prediction models for all three coagulation traits were below the threshold to consider the prediction models adequate even for rough screening of the samples.

## 1. Introduction

Water buffalo are rustic, long-living animals (with up to 20 years of productive life) that produce, on average, between 600 and 4500 L of milk per lactation [1]. Compared to cow milk, buffalo milk presents greater fat, protein, casein, lactose, minerals, and total solids [1,2] and has additional health benefits due to their anti-inflammatory, antioxidant, and anticarcinogenic properties [1]. India has the largest water buffalo (*Bubalus bubalis*) population in the world, accounting for 55% of the global population and producing 71% of total buffalo milk [3]. On the other hand, Italy is the largest producing European country in terms of animal, milk, and cheese production, accounting for 86%, 88%, and 57%, respectively, within the European Union [3]. Moreover, Italy is the second biggest buffalo cheese manufacturer worldwide after Egypt [3]. In the Italian dairy industry, the assessment of buffalo milk coagulation properties is of utmost importance as it is predominantly transformed into ‘Mozzarella di Bufala Campana’ Protected Designation of Origin (PDO), where Campania and Lazio regions concentrate 90% of Italian buffalos [1]. Other buffalo milk- and whey-cheeses such as ‘burrata di bufala’ and ‘buffalo ricotta’ are also produced [1].

The Formagraph mechanical system is commonly used to determine milk coagulation properties, including milk rennet coagulation time (RCT), curd firming time (k_20_), and curd firmness 30 min after rennet addition to milk (**a_30_**) [4]. However, this method cannot be used as a process of analytical control and monitoring of milk rennetability because it is time-consuming and allows only for a few samples to be analyzed within 1 h. On the other hand, mid-infrared (MIR) spectroscopy is commonly used to predict milk gross composition during the official milk controls, including fat, protein, casein, and lactose. It also gives the possibility to record the spectra obtained to apply future prediction models. This technique is cost-effective and easy to use, allowing for rapid determination of multiple parameters. Recently, MIR has also been used as an authentication method to detect buffalo milk adulteration with high accuracy to distinguish it from cow milk [5,6,7].

Studies conducted with the individual milk of goats [8] and sheep [9] and with bulk milk from cow herds [10] have revealed the low accuracy of the prediction models for milk coagulation properties. To our knowledge, only one study conducted in 2017 has focused on its applicability to predict the coagulation properties of individual milk samples [11]. This study collected individual samples from a single milking, taking into consideration the individual variability of the traits, including detecting samples that do not coagulate within 30 min of the coagulation analysis [11]. Despite the MIR prediction models revealing a low predictive ability for RCT, k_20_, and a_30_, it correctly identified the noncoagulating samples [11]. However, the milk payment system is based on bulk milk that includes the complete milk production of a herd from two milkings to be representative of the daily milk production and not individual samples. Thus, it is necessary to confirm the results obtained with individual milk.

Therefore, this study aimed to evaluate the feasibility of MIR spectroscopy for predicting coagulation properties (RCT, k_20_, and a_30_) of water buffalo bulk milk.

## 2. Materials and Methods

### 2.1. Bulk Milk Sampling and Analysis

A total of 1736 bulk milk samples (60 mL without preservative) of Mediterranean Italian buffaloes were collected from 55 farms located in the Lazio region of central Italy between 2021 and 2023. This is the area of Mozzarella di Bufala Campana PDO. Bulk milk samples were obtained from 2 consecutive milkings (morning and evening) and transported refrigerated (4 °C). They were analyzed within 36 h of collection at the quality milk laboratory Istituto Zooprofilattico Sperimentale del Lazio e della Toscana “M. Aleandri” (Rome, Italy), which is accredited by Accredia, the Italian Accreditation Body (Laboratory number 0201A), and follows International Organization for Standardization ISO/IEC 17025:2017. Milk chemical composition (i.e., fat, protein, casein, and lactose content) was determined with MilkoScan^TM^ 7 RM (Foss Analytical A/S, Hillerød, Denmark), which is calibrated with appropriate buffalo standards. The somatic cell count (SCC) was assessed with a Fossomatic FC system (Foss Electric, Hillerød, Denmark).

The reference values for milk coagulation traits (RCT, k_20_, and a_30_) were obtained using a Formagraph LDG 2.0 (Ma.Pe System srl, Firenze, Italy). To obtain these values, milk samples (10 mL) were initially heated to 36 °C, and 200 µL of calf rennet (comprising 75% chymosin and 25% pepsin, 175 international milk clotting units/mL; Clerici s.p.s., Sacco srl, Cadorago, Italy) diluted to a concentration of 1% (*wt*/*wt*) in distilled water were added. Measurement concluded 30 min after the enzyme addition.

### 2.2. Chemometric Analysis

Spectral information of bulk milk collected during the gross composition determination using the MilkoScan^TM^ 7 RM (Foss Electric, Hillerød, Denmark) was recorded as a log (1/Transmittance). The instrument works within the range of 5000 to 900 cm^−1^, providing 1060 data points. To develop the prediction models, the spectral information was matched with reference values for milk coagulation traits. Prediction models were built with WinISI 4 software (Infrasoft International, Port Matilda, PA) through modified partial least-squares regression analysis (mPLS) [12] after removing the noisy areas related to water (1566 to 1712 cm^−1^; 1817 to 2696 cm^−1^; 2975 to 500 cm^−1^. The mPLS is considered more accurate than the PLS and the standard method to develop the prediction models with WinISI software [13]. Any spectral outliers were removed based on the Mahalanobis distance (Global H > 3.0), followed by 3 rounds of chemical outliers’ elimination using the T-statistic (T > 3.0). Moreover, 58 milk samples (3.38%) did not clot within the 30-min test period and were also discarded from the chemometric analysis. The raw spectra were then subjected to several scatter corrections (D, detrending; SNV, standard normal variate; SNV+D; MSC, multiplicative scatter correction) to reduce noise and remove imperfections combined with mathematical treatments (0,0,1,1; 1,4,4,1; 1,8,8,1; 2,5,5,1; 2,10,10,1; where the first digit is the number of the derivative, the second one is the gap over which the derivative is calculated, the third one is the number of data points in the first smoothing, and the fourth one is the number of data points in the second smoothing) [14]. In more detail, scatter is a nonlinear function that can distort the relationship between the NIR spectrum and the reference value. In WinISI software, five options are available: SNV scales each spectrum to have a standard deviation of 1.0 to help reduce the effects of particle size. Detrending removes the linear and quadratic curvature of each spectrum. The SNV+D allows us to evaluate SNV and D together. The MSC uses a correction for mean and standardization at each wavelength. These five methods are the most widely used and efficient for testing to improve calibration accuracy.

The dataset was then split into a calibration set (75% of the observations) and a validation (25% of the observations) set using a random selection method to ensure similar mean and standard deviation (SD) values for each trait across both sets. Calibration models were developed using an iterative 15-fold cross-validation and then tested in the validation set. The performance of the prediction models was assessed using the number of latent factors (LF), the standard error of cross-validation (SEC), the coefficient of determination in cross-validation (R^2^_CrV_), the standard error in external validation (SEP), the coefficient of determination in external validation (R^2^_ExV_) and the residual prediction deviation (RPD), the bias, and the slope. The RPD was calculated as the dataset’s SD divided by the SEP, and the bias was calculated as the difference between the predicted and the reference data. The SEP is considered a true indication of the performance of the equation on unknown samples from the same population [15]. The interpretation of R^2^ and RPD were as follows: equations with R^2^_ExV_ < 0.66 and RPD < 0.75 are not recommended, R^2^_ExV_ between 0.66 and 0.81 and RPD between 1.7 and 2.2 are adequate for screening proposes, R^2^_ExV_ between 0.83 and 0.90 and RPD between 2.3 and 3.5 should be used with caution, R^2^_ExV_ between 0.92 and 0.96 and RPD between 3.6 and 4.9 are adequate for most applications, and R^2^_ExV_ > 0.98 and RPD > 5.0 are adequate for any application [16]. Bias should be closer to 0 and slope closer to 1.

## 3. Results and Discussion

### 3.1. Descriptive Statistics

Table 1 displays the characteristics of both the calibration and validation datasets. Both datasets have comparable means and SD and cover a similar range for all the analyzed traits, which is important for developing reliable infrared calibration models [17]. The chemical composition of the evaluated milk samples was consistent with previous studies in the same area with bulk [4] and individual [11] buffalo milk. Moreover, these results are in line with a comprehensive review of the nutritional value and technological properties of milk from several dairy species, including buffalo [18].

The 3.38% of the samples did not coagulate within the analysis. Other authors have also reported a greater presence of noncoagulating samples when dealing with individual milk from buffalo (16.9%) [11], sheep (12.9%) [9], and goats (7.9%) [8]. Regarding buffalo milk coagulation properties (Table 1), a previous study reported a shorter RCT (13.14 min) but a longer k_20_ (4.89 min) with a thicker a_30_ (48.32 mm) in bulk milk [4]. In individual buffalo milk samples, the same authors reported similar k_20_ (3.17 min) and a_30_ (39.52 mm) but a shorter RCT (13.33 min) [11]. Nevertheless, the current results for milk coagulation traits were within the range reported by these two previous studies [4,11]. On the other hand, other authors have reported a shorter RCT (8.46 min) and k_20_ (0.98 min) but a thicker a_30_ (41.32 mm) with individual buffalo milk [19].

### 3.2. Prediction Models Performance

**Figure 1:**
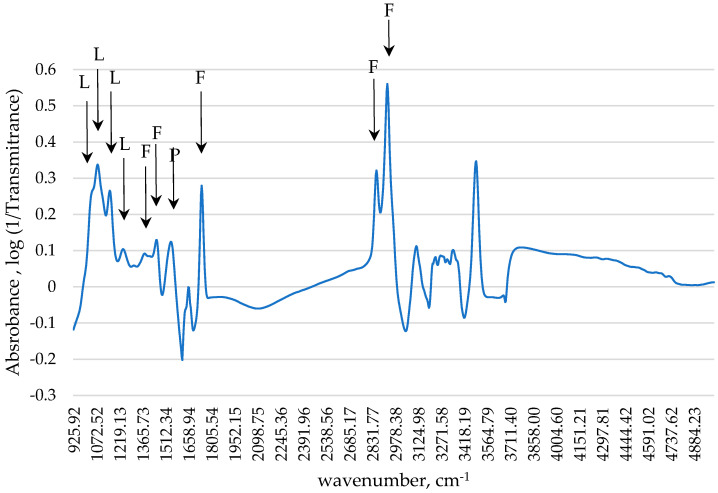
Mid-infrared average raw spectra of buffalo bulk milk samples. Abbreviations: L, Lactose; P, Protein; F, Fat.

Figure 1 shows the average raw spectrum of the milk sample. This spectrum is similar to cow [20] and sheep milk spectra [9]. The peak observed around 1045 cm^−1^ corresponds to the C–O stretching vibration of alcohol functions, at 1076 cm^−1^ to C–O, C–C, and C–H stretching vibration, and 1157 and 1250 cm^−1^ with C–O–C ether stretching [20]. It has been described that these peaks are related to lactose content [20]. Peaks around 1550 cm^−1^ correspond to C–N and N–N stretching, which is linked to protein content [20]. Peaks around 1390 and 1454 cm^−1^ correspond to C–H bending of −CH_3_ and −CH_2_, around 2862 and 2927 cm^−1^ to C–H stretching of −CH_3_ and −CH_2_, and around 1743 cm^−1^ to the C=O ester stretching [20]. It has been described that these peaks are related to fat content [20].

The percentage of spectral and chemical outliers removed before building the calibration models was 4.36% for RCT, 6.16% for k_20_, and 4.84% for a_30_. Although this proportion is within the accepted range (<10%), it is greater than the <2% reported previously for individual buffalo milk samples [11]. Among all the scatter corrections, the best prediction models for RCT and a_30_ were obtained by applying the SNV correction, whereas the best prediction model for k_20_ was achieved with D. Despite testing the first and second derivatives as mathematical treatments, the best prediction models for k_20_ and a_30_ were obtained without mathematical derivation of the raw absorbance. On the other hand, the best prediction model for RCT was achieved when using the first derivative.

The LF retained for the final calibration models is displayed in Table 2. The k_20_ shows the lowest number of LF among all the parameters, whereas a_30_ is the greatest one, reaching 13 LF. The greater number of LF for RCT and a_30_ indicates that the calibration models present some difficulties in accurately predicting these parameters [21]. In individual buffalo milk samples, a greater LF (between 15 and 17) was needed to achieve a similar prediction performance as the one reported in Table 2 [11]. In cow milk, a greater number of LF (15 LF) was also selected than reported in Table 2 to achieve a similar performance of the models in the calibration set [10]. By reducing the number of LF in an infrared prediction model, the potential for overfitting can be reduced, which can improve the prediction model’s generalization to new data [22]. Thus, our models could be considered slightly better than the ones in the literature in terms of overfitting.

The bias of the prediction models was found to be near zero (Table 2). However, upon examining the slope of the prediction models, it was observed that the models could be considered less precise at the extreme ends of the range covered, as the slope deviated ± 0.15 from the unity (0.85–1.15; [21]). Prediction models are considered adequate when the slope deviation is between 0.95 and 1.05 [23].

Similar R^2^_CrV_ and R^2^_ExV_ were found, indicating that the samples were correctly divided into calibration and validation sets. Among all three milk coagulation traits evaluated, the best prediction model was built for a_30_. On the other hand, RCT and k_20_ performed similarly in terms of R^2^_ExV_ and RPD; however, the values were below 0.41 and 1.30, respectively. Based on these statistics, the prediction models were insufficient for their implementation as R^2^_ExV_ and RPD were <0.66 and <0.75, respectively [16]. The prediction models were slightly better than the previous results with individual buffalo milk samples, where the best model was reached for a_30_ with an R^2^_ExV_ of 0.35 and RPD of 1.20 [11].

Moreover, these results are consistent with the use of MIR spectroscopy to predict milk coagulability across different types of milk. For instance, goat milk MIR prediction models developed with individual milk samples also reported a low R^2^_ExV_ for all three traits [8]. While the R^2^_ExV_ for RCT was similar (0.42) to the one we obtained (Table 2), their prediction models achieved a lower k_20_ and a_30_ (0.29 and 0.27, respectively) [8]. In individual sheep milk samples, the R^2^_ExV_ for RCT was slightly greater (0.39), but the ones for k_20_ and a_30_ (0.37 and 0.31, respectively) were lower compared to our results (Table 2) [9]. In cow bulk milk, the prediction models were slightly better using a greater number of LF (15 LF), reaching an R^2^_CrV_ of 0.65 for RCT, 0.49 for k_20_, and 0.68 for a_30_ [10].

## 4. Conclusions

In conclusion, MIR prediction models for coagulation traits in buffalo milk were consistent with previous models created for individual buffalo milk and other milk-producing animals like cows, sheep, and goats. The developed prediction models for all three milk coagulation properties slightly improved their accuracy compared with the ones from individual buffalo milk samples. However, these models were still below the threshold for the prediction models to be considered adequate even for a rough screening of the samples.

## Figures and Tables

**Table 1:** Descriptive statistics ^1^ of milk coagulation traits and chemical composition in calibration and validation sets for Mediterranean buffalo bulk milk.

Trait ^2^	N	Mean	SD	CV	Minimum	Maximum
Calibration set						
RCT, min	1259	17.71	3.75	21.18	7.37	29.45
k_20_, min	1168	3.29	1.14	34.52	0.37	7.15
a_30_, mm	1260	38.83	14.01	36.09	0.98	70.20
Fat, %	1281	7.87	1.19	15.08	3.99	11.86
Protein, %	1302	4.64	0.35	7.64	3.15	7.53
Casein, %	1296	3.68	0.36	9.83	2.54	4.89
Lactose, %	696	4.64	0.18	3.87	3.69	5.18
SCC, cell/µL	1302	181.54	274.08	150.98	11.00	3486.00
Validation set						
RCT, min	419	17.71	3.71	20.94	8.00	29.30
k_20_, min	389	3.31	1.13	34.05	1.15	7.15
a_30_, mm	420	38.92	13.96	35.86	2.00	73.84
Fat, %	426	7.87	1.17	14.92	4.19	11.75
Protein, %	434	4.64	0.37	7.89	3.57	7.53
Casein, %	431	3.68	0.36	9.72	2.63	4.81
Lactose, %	231	4.64	0.17	3.70	4.03	5.06
SCC, cell/µL	433	178.07	251.58	141.28	16.00	2734.00

^1^ SD, Standard Deviation; CV, Coefficient of Variation. ^2^ RCT, Rennet Coagulation Time; k_20_, curd-firming time; a_30_, curd firmness at 30 min after rennet addition to milk; SCC, somatic cells count.

**Table 2:** Fitting statistics ^1^ of prediction models based on a modified partial least-squares regression for bulk milk coagulation traits ^2^ for Mediterranean buffalo using Fourier-transform mid-infrared spectroscopy.

	Calibration Set						Validation Set					
Trait	N	Scatter Correction	Mathematical Treatment	LF	SEC	R^2^_CrV_	N	Bias	Slope	SEP	R^2^_ExV_	RPD
RCT, min	1204	SNV	1,4,4,1	11	2.83	0.40	419	0.03	0.92	2.90	0.40	1.29
k_20_, min	1096	Detrend	0,0,1,1	6	0.82	0.39	389	0.07	1.08	0.88	0.41	1.30
a_30_, mm	1199	SNV	0,0,1,1	13	8.60	0.61	420	0.02	0.93	9.08	0.57	1.52

^1^ N, number of samples; SNV, standard normal variate; LF = latent factors; SEC = standard error of prediction of cross-validation; R^2^_CrV_, coefficient of determination of cross-validation; SEP, standard error of prediction of external validation; R^2^_ExV_ = coefficient of determination of external validation; RPD = ratio of prediction to deviation calculated as the ratio between the standard deviation of the trait and the SEP. ^2^ RCT, Rennet Coagulation Time; k_20_, curd-firming time; a_30_, curd firmness at 30 min after rennet addition to milk.

## Data Availability

The original contributions presented in the study are included in the article, further inquiries can be directed to the corresponding author.
